# Copy Number Variation Identification on 3,800 Alzheimer’s Disease Whole Genome Sequencing Data from the Alzheimer’s Disease Sequencing Project

**DOI:** 10.3389/fgene.2021.752390

**Published:** 2021-11-04

**Authors:** Wan-Ping Lee, Albert A. Tucci, Mitchell Conery, Yuk Yee Leung, Amanda B. Kuzma, Otto Valladares, Yi-Fan Chou, Wenbin Lu, Li-San Wang, Gerard D. Schellenberg, Jung-Ying Tzeng

**Affiliations:** ^1^ Penn Neurodegeneration Genomics Center, Department of Pathology and Laboratory Medicine, Perelman School of Medicine, University of Pennsylvania, Philadelphia, PA, United States; ^2^ Institute for Biomedical Informatics, Perelman School of Medicine, University of Pennsylvania, Philadelphia, PA, United States; ^3^ Department of Pathology and Laboratory Medicine, Perelman School of Medicine, University of Pennsylvania, Philadelphia, PA, United States; ^4^ Bioinformatics Research Center, North Carolina State University, Raleigh, NC, United States; ^5^ Division of Human Genetics, Children’s Hospital of Philadelphia, Philadelphia, PA, United States; ^6^ Graduate Group in Genomics and Computational Biology, Perelman School of Medicine, University of Pennsylvania, Philadelphia, PA, United States; ^7^ Department of Statistics, North Carolina State University, Raleigh, NC, United States

**Keywords:** copy number variation—CNV, Alzheiemer’s disease, whole-genome sequence (WGS), CNV association test, NGS—next generation sequencing

## Abstract

Alzheimer’s Disease (AD) is a progressive neurologic disease and the most common form of dementia. While the causes of AD are not completely understood, genetics plays a key role in the etiology of AD, and thus finding genetic factors holds the potential to uncover novel AD mechanisms. For this study, we focus on copy number variation (CNV) detection and burden analysis. Leveraging whole-genome sequence (WGS) data released by Alzheimer’s Disease Sequencing Project (ADSP), we developed a scalable bioinformatics pipeline to identify CNVs. This pipeline was applied to 1,737 AD cases and 2,063 cognitively normal controls. As a result, we observed 237,306 and 42,767 deletions and duplications, respectively, with an average of 2,255 deletions and 1,820 duplications per subject. The burden tests show that Non-Hispanic-White cases on average have 16 more duplications than controls do (*p*-value 2e-6), and Hispanic cases have larger deletions than controls do (*p*-value 6.8e-5).

## Introduction

Alzheimer’s disorder (AD) is a devastating neurodegenerative disease and is the most common cause of dementia. Approximately 6.2 million Americans are living with AD in 2021, and it is projected to reach 12.7 million in 2050, which makes AD one of the most pressing public health issues (Alzheimer’s Association, 2020). Presently, there is no known effective prevention or disease modifying therapies, and the landscape of AD drug trials is gloomy. One possible reason is that AD is a heterogeneous disorder, but trials are designed treating it as a monolithic disease. Although lifestyle and environmental risk factors clearly affect AD, the primacy of genetic influences suggests that categorization by genetic basis should be prioritized in developing effective interventions.

AD heritability estimates range from 49–79%; however, <50% of this heritability can be explained by genome-wide association studies (GWAS) on single nucleotide variants (SNVs) (Ridge et al., 2013; Sims et al., 20202020). Taking copy number variation (CNV) into consideration may partially mitigate the problem of missing heritability and play an important role in human disease susceptibility (Cooper et al., 2011; Chung et al., 2014; McCarroll and Altshuler, 2007; Kakinuma and Sato, 2008; Cooper et al., 2011; Chung et al., 2014; McCarroll and Altshuler, 2007; Kakinuma and Sato, 2008). For neuropsychiatric disorders, such as intellectual ability, Autism Spectrum disorders, Schizophrenia, and Bipolar disorder, CNVs have given rise to a new understanding of disease etiology (Kakinuma and Sato, 2008; Malhotra and Sebat, 2012; Sullivan et al., 2012). Recently, multiple studies have highlighted the roles of CNVs in AD as well (Szigeti et al., 2013; Szigeti et al., 2014; Saykin et al., 2011; Heinzen et al., 2010; Lew et al., 2018; Zheng et al., 2015; Zhang, 2020; Heinzen et al., 2010; Saykin et al., 2011; Szigeti et al., 2013; Szigeti et al., 2014; Zheng et al., 2015; Lew et al., 2018; Zhang, 2020). For example, an intragenic CNV is found in the *CR1* gene (Brouwers et al., 2012), and people with Down syndrome have a higher chance to develop neuropathology, consistent with the observation that AD may be caused by duplications in the *APP* gene in chromosome 21 (Goate, 2006; Lanoiselée et al., 2017). However, there is no comprehensive genome-wide CNV study using whole-genome sequence (WGS) to enhance the knowledge of AD etiology and risk.

Most of the previous CNV GWAS of AD were performed using genotyping array data. Although these arrays can quickly and cost efficiently genotype large numbers of samples, there are serious technological limitations in that only large CNVs spanning multiple pre-determined probes can be reliably detected. However, WGS data allows an unbiased investigation of CNVs of all types (i.e., small and large; common and rare; within coding and non-coding regions) and provides a unique opportunity to comprehensively study CNVs in diseases. To accelerate AD genetic discovery, the Alzheimer’s Disease Sequencing Project (ADSP) (Beecham et al., 2017), a strategic program funded by the National Institute on Aging (NIA), is committed to sequence AD cases, and cognitively normal elder controls from multi-ethnic populations, providing a valuable resource for genome-wide identification of CNVs.

This study utilizes the ADSP Umbrella R1 dataset (ng00067) released through the National Institute on Aging Genetics of Alzheimer’s Disease Data Storage Site (NIAGADS) Data Sharing Service (Kuzma et al., 2016). After quality and relatedness checks, we had 1,737 AD cases and 2,063 cognitively normal elder controls for this study. We employed three CNV calling algorithms, CNVnator (Abyzov et al., 2011), JAX-CNV (Lee et al., 2021), and Smoove (GitHub—brentp/smoove, 2021; Layer et al., 2014) that on average detected 2,378, 25, and 4,584 CNVs, respectively, for each sample. GraphTyper2 (Eggertsson et al., 2019) was then applied for joint genotyping to generate a single VCF for all 3,800 samples in the study, which increased the number of CNVs to 280,073 average/sample; however, most of those CNVs either overlap or are adjacent to each other. After merging CNVs of the same type (deletions or duplications) and removing conflict regions with different types of CNVs, there are on average 4,075 CNVs per sample. The CNVs we identified tended to be more abundant and longer in AD cases compared to cognitively normal, elder controls, though in most cases this trend was not statistically significant.

## Materials and Methods

The analysis flow consists of two major steps; identification of CNVs from WGS from 3,800 subjects (*CNV Identification on WGS Data*), and CNV burden analysis (*CNV Burden Analysis Using PLINK*).

**FIGURE 1:**
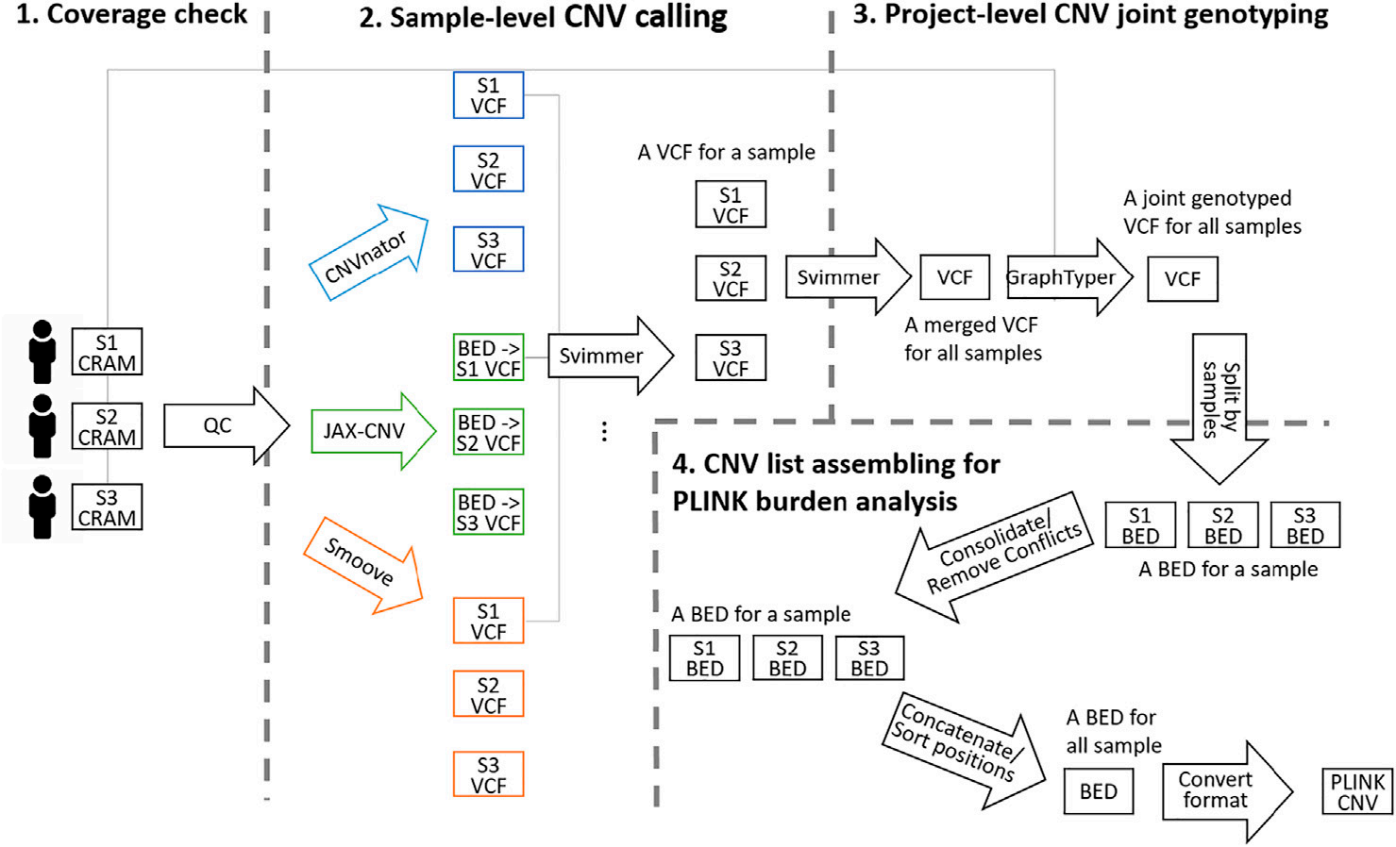
Overview of the CNV identification workflow from WGS data consisting of the four steps. **1)** Alignment coverage check. **2)** Sample-level CNV calling including calling by CNVnator, JAX-CNV and Smoove, and merging the three callsets by Svimmer. Since Svimmer takes the VCF format as input, results of JAX-CNV in the BED format were converted to the VCF format. **3)** Project-level CNV re-genotyping. **4)** CNV list assembling for PLINK burden analysis. The illustrated three samples in the figure are notated by S1, S2 and S3 while 3,800 samples were processed in the study.

Figure 1 shows an overview of the flow of CNV identification on WGS data. The flow starts with alignment CRAM files and ends at the single-sample CNV list generation. The process began with a quality check (WGS Across-Chromosome Coverage Check) followed by sample-level CNV calling and project-level CNV joint genotyping (Sample-Level CNV Calling and Project-Level CNV Joint Genotyping). Finally, to meet the data format requirements of CNV burden analysis, the genotyped VCF was further split as a list in BED format per sample for region consolidation (for same-type CNVs overlapping) and removal (for different-type CNVs overlapping). Then, all BED files were merged and converted in PLINK format as the input of burden analysis (CNV List Assembling for PLINK Burden Analysis). The detailed scripts are given in supplementary material.

### CNV Identification on WGS Data

#### WGS Across-Chromosome Coverage Check

The quality of CNV calling on WGS data is sensitive to alignment coverages across all chromosomes of a sample. Uneven coverages of chromosomes may cause false positive CNVs. Thus, before calling CNVs, it is necessary to perform a quality check of alignment coverages. Samples with uneven coverage were removed from analysis.

We developed a method (implemented as part of JAX-CNV) to first estimate the coverage of each chromosome of a sample. The method seeks 20 repetitive-free regions in each chromosome, and then calculates an average coverage of these regions to present the coverage of the chromosome. A repetitive-free region is defined as a 20k bp long region with each 25-mer (k-mer) inside the region having a unique position in the entire reference genome.

Once coverage of each chromosome was obtained, we were able to identify outlier chromosomes with unexpected high or low coverages. For example, outliers could indicate trisomy, monosomy, and other gross chromosome number anomalies. An overall average coverage of a sample was then computed by using the coverages of all chromosomes excluding outliers. A standard deviation of chromosomes coverages was employed as the metric to identify problematic samples that were removed from downstream analyses. This method is fast and takes approximately 5 minutes for a 30X sequence sample.

#### Sample-Level CNV Calling

We employed CNVnator, JAX-CNV, and Smoove for CNV detection. CNVnator and JAX-CNV are Read-Depth-based (RD-based) algorithms while Smoove utilizes multiple signals of RD, Paired-End (PE), and Split-Read (SR). CNVnator is sensitive for CNVs sizes ranging from 1 to 50 kb; however, it may break larger CNVs into smaller pieces that introduce difficulties for downstream analyses. We included JAX-CNV in the analysis flow because it was developed to detect large (>50 kb) CNVs and resolves the issue of fine pieces from CNVnator. Smoove was recruited to strengthen small CNV (<1 kbp) identification. These three CNV calling algorithms are not only fast but also generating high-quality CNVs. Moreover, the combination of them allows us to cover the complete size spectrum of CNVs.

For each sample, we applied these three algorithms separately. Each algorithm could generate a BED (JAX-CNV) or VCF (CNVnator and Smoove) file to store a set of deletions/duplications with genomics coordinates and genotypes (homozygous or heterozygous, and copy numbers) of a sample. If a BED file was generated, we converted it to VCF format to facilitate the step of utilizing svimmer (GitHub—DecodeGenetics/svimmer, 2021) for callset merging. For variant types (deletions, duplications, inversions, and breakends) detected by Smoove, we only kept deletions and duplications. For each sample, we then applied svimmer to merge the three VCFs obtained from the three algorithms.

#### Project-Level CNV Joint Genotyping

Joint analysis is recommended for a dataset with multiple samples. Once variants of a sample were detected, a joint analysis step provides the ability to leverage population-wide information from multiple samples that allows us to refine low-quality genotypes and detect additional variants of a sample. For example, a joint genotyping step is suggested in the GATK best practice for SNV and INDEL detection.

Compared to SNV/INDEL joint genotyping, CNV joint genotyping is challenging since breakpoints of CNVs from short-read sequence data may be imprecise. By incorporating detected variants within the linear reference genome, the emerging methodology, Graph Genome, provides a good model for joint genotyping CNVs across multiple samples in a single step. We evaluated GraphTyper2 (Eggertsson et al., 2019), Paragraph (Chen et al., 2019), and VG (Hickey et al., 2020), and selected GraphTyper2 in the analysis flow due to its balance of required computational resource and quality of results.

As GraphTyper2 recommended, we employed svimmer (GitHub—DecodeGenetics/svimmer, 2021) to merge all sample-level VCFs and generate a single VCF that does not contain genotypes. GraphTyper2 was then applied on this merged VCF with all CRAM files for each 500kb region excluding the centromeres. GraphTyper2 generated a VCF of CNVs with genotypes of all samples. There are three models used for joint genotyping in GraphTyper2, Aggregated, Coverage, and Breakpoint, and we kept results from Aggregated model as GraphTyper2 suggests. We also applied PASS flag filter in the GraphTyper2 VCFs. Each 500kb chunk VCFs were consented using BCFtools (Danecek et al., 2021).

### CNV Burden Analysis Using PLINK

#### CNV List Assembling for PLINK Burden Analysis

There remains a challenge in using GraphTyper2 VCF files for downstream burden analysis. Since multiple calling algorithms were applied for CNV identification, CNV lengths and breakpoints may vary. Although GraphTyper2 was applied to mitigate this situation, we still can find CNV segments overlapping each other that is not acceptable by downstream association analysis tools such as PLINK (Chang et al., 2015). To resolve overlapping segments, we first split CNVs (with PASS genotype tags) of a sample in BED format for each sample. The BED is in the format of chromosome, begin position, end position, and copy number status for each CNV. The copy number status recorded as 0, 1, 3 or 4 copies. Of note, the copy status 4 includes copy numbers equal or larger than 4. Then, we used BEDTools (Quinlan and Hall, 2010) to merge overlapping or adjacent segments. Segments were merged only if they are the same CNV type, deletions or duplications. For those regions having different CNV types, we filtered them out since the downstream association analysis would not take those regions into consideration. Once the CNV consolidation and removal were done for all samples, we then concatenated all BED files and sorted the merged BED file by CNV positions.

PLINK format, that is commonly accepted by other downstream association tools, is a tabular file format with CNV coordinates, family IDs, and sample IDs. Since there are no related samples in the dataset, we replicated sample IDs as family IDs. We then converted the BED file into a six-column with family ID, sample ID, chromosome, start position, end position, and copy number status, e.g. 0, 1, 3, or 4 copies.

#### Rare CNV Identification

Rare CNVs were obtained using PLINK to impose a 0.01 frequency threshold (i.e., --cnv-freq-exclude-above 38 and--cnv-overlap 0.5), which removed CNVs with >50% of its length spanning a region with >1% × 3,800 CNVs in the dataset. The same approach was applied on African American (AA) (--cnv-freq-exclude-above 9), Hispanic (--cnv-freq-exclude-above 12), and Non-Hispanic White (NHW) (--cnv-freq-exclude-above 15) samples. Then, we applied the pilot mask released by the 1,000 Genomes Project (The 1000 Genomes Project, 2010) on rare CNV lists. The pilot mask was done by looking at the amount of sequence data that aligned to any given location in the reference genome. Regions are defined inaccessible if their depth of coverages (summed across all samples in the 1,000 Genomes Project) were higher or lower than the average depth. The mask results in 5.3% of bases marked “N” (the base is an “N”), 1.4% marked “L” (coverage is low), 0.6% marked “H” (coverage is high) and 3.7% marked “Z” (many reads mapped here have zero quality). The remaining 89.0% of are marked “P” (regions are good and passed). All rare CNVs need to reside in “P” regions.

#### CNV Burden Analysis

We examined the burdens of all and rare CNVs in AD cases and controls using PLINK. PLINK burden analysis uses permutation tests to compute *p*-values. For our analysis, we applied 500,000 permutations. For each sample, we considered four CNV burden features: 1) number of CNV events; 2) proportion of samples with ≥1 CNV events; 3) total event length in kb; and 4) average event length in kb. The CNV events included deletions and duplications together (DelDup), deletions specific (Del), and duplications specific (Dup). We reported the CNV burdens for AA, Hispanic, and NHW separately as well as for all-combined samples (ALL), The Bonferroni threshold for multiple testing is *p*-value < 0.05/96 analyses = 0.000521, where the 96 analyses included the combinations from 2 sets of CNV analyses (all CNVs vs. rare CNVs), 4 burden features, 3 CNV events (DelDup, Del, and Dup) and 4 sample groups (ALL, AA, Hispanic, and NHW).

## Results

### Dataset—3,800 WGS Samples from NIAGADS R1 Release of ADSP 5k

We used the ADSP WGS data released by NIAGADS in 2018. NIAGADS not only collected and released genetics data, but also harmonized minimal phenotypes (sex, race/ethnicity, diagnosis, *APOE* genotype) from each collocating cohort. For data harmonization, NIAGADS followed the ADSP coding scheme based on the National Alzheimer’s Coordinating Center (NACC) Uniform Data Set (UDS) (Beekly et al., 2007) definitions. We used NIAGADS and did not redefine diagnosis or ethnicities in this study.

There are 4,749 subjects and 4,788 sequenced samples (three subjects sequenced nine times and another three sequenced six times) by Illumina HiSeq 2000/2,500 or X Ten at an average of 37X coverage (the range from 10.68X to 74.16X). For the six subjects with multiple sequence sets, we picked one sequence set per subject, and removed the other 39 sequences. For the 4,749 subjects, these were 2,192 AD cases, 2,073 controls, and others 484 with diagnosis unknowns. For this study, we focused on AD cases and controls, and excluded samples with inconclusive clinical statuses.

**FIGURE 2:**
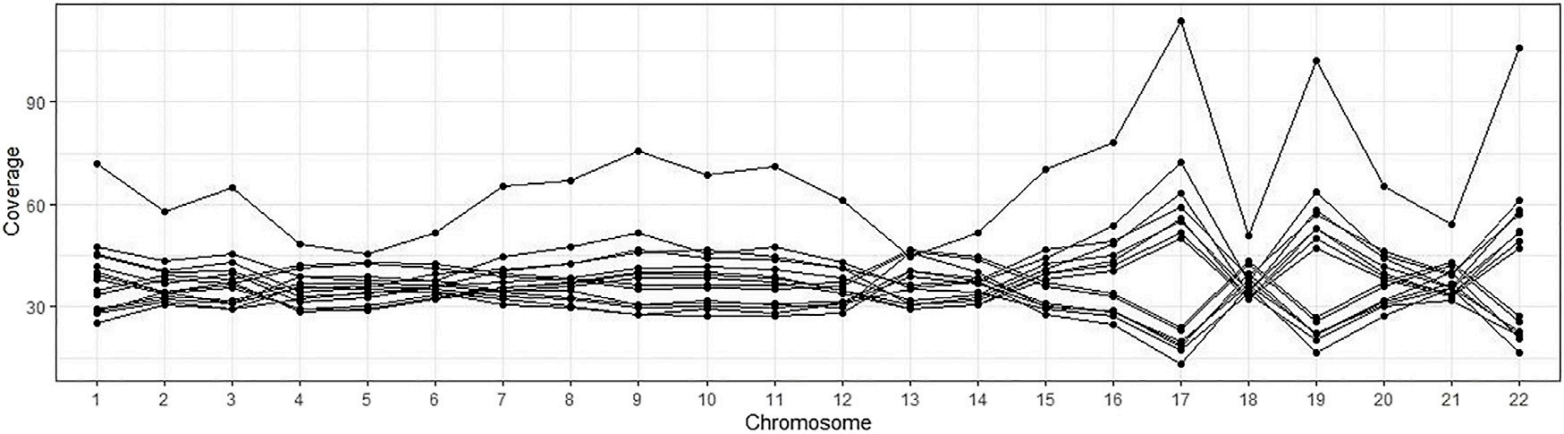
Alignment coverages of 15 samples with uneven sequence data. Each line is a sample, and each dot presents the alignment coverage for a chromosome.

For the remaining 4,265 samples, we performed the across-chromosome alignment coverage check (*WGS Across-Chromosome Coverage Check*) since uneven coverage may affect the quality of CNV detection. Fifteen samples were removed since their standard deviation of chromosomes coverages are greater than 15% of the average coverages, as shown in Figure 2 where each line presents a sample, and each dot presents the alignment coverage of the sample in the chromosome on the *x*-axis.

Next, we removed 450 samples due to relatedness according to pedigree information provided by NIAGADS. Finally, we had 1,737 AD cases and 2,063 controls. The ethnicities/races are AA (*n* = 978), Hispanic (*n* = 1,247), NHW (*n* = 1,566), and others (*n* = 9), as shown in Table 1.

**TABLE 1:** Total column denotes the number of samples remaining after each quality filtering step.

	AA			Hispanic			NHW			Others			Total
**Step**	**Case**	**Control**	**Unknown**	**Case**	**Control**	**Unknown**	**Case**	**Control**	**Unknown**	**Case**	**Control**	**Unknown**	
ADSP 5K	472	521	44	826	746	40	910	820	393	5	4	7	4,788
Replicate Removal	467	521	44	810	733	40	910	815	393	5	4	7	4,749
Unknown Status Removal	467	521	0	810	733	0	910	815	0	5	4	0	4,265
Uneven Coverage Removal	466	521	0	808	731	0	902	813	0	5	4	0	4,250
Relatedness Removal	457	521	0	520	727	0	755	811	0	5	4	0	**3,800**

3,800 samples remained after all filtering steps.

### CNV Callset

**FIGURE 3:**
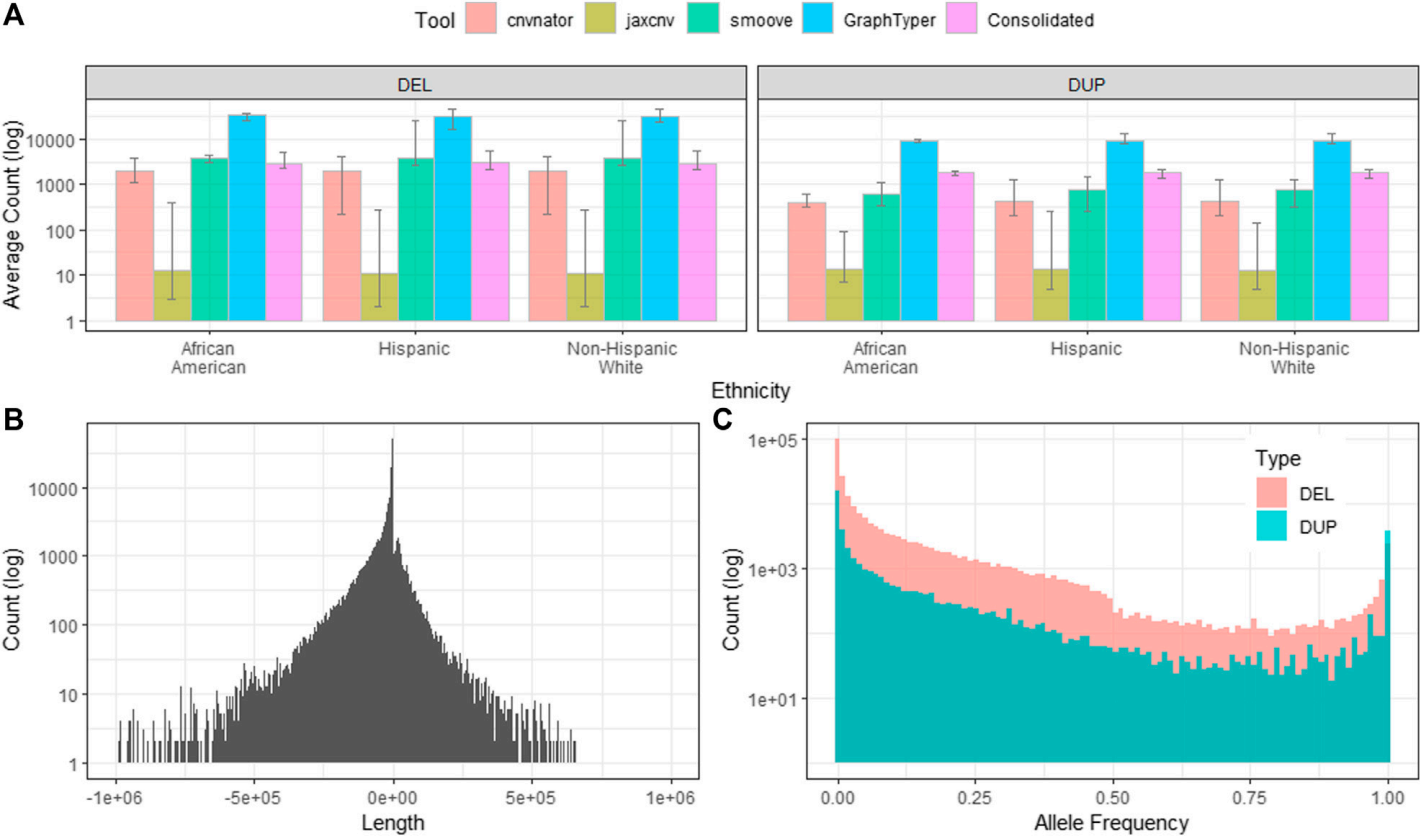
Characteristics of the project level CNV callset. Counts shown on the *y*-axes of the sub figures are in the log10 scale. **(A).** The average deletions and duplications detected by CNVnator, JAX-CNV, Smoove and GraphTyper2. Consolidated shows CNV counts after CNV merging and conflicts removing. **(B).** Length distribution of CNVs after applying GraphTyper2 and the PASS flag filter. Lengths of deletions were presented as negative values while lengths of duplications are positive values. **(C).** Allele frequency of CNVs of GraphTyper2.

We first applied CNVnator, JAX-CNV and Smoove on each CRAM file of a sample for sample-level CNV calling. CNVnator, JAX-CNV and Smoove detected an average of 2,378 (1,967 deletions and 411 duplications), 25 (12 deletions and 13 duplications), and 4,584 (3,876 deletions and 708 duplications) CNVs, respectively. Compared to NHW, AA and Hispanic have 141 and 122 deletions more, but 180 and 9 fewer duplications. Only Smoove yielded fewer duplications for AA and Hispanic, as shown in Figure 3A.

For each sample, we employed svimmer to merge the callsets from the three callers as a single VCF. Next, svimmer was applied to VCFs for all 3,800 samples to generate a combined VCF which along with all CRAM files are inputs of GraphTyper2. As described in *Project-Level CNV Joint Genotyping*, we kept Aggregated notated variants and also applied the PASS flag filter in this aggregated callset. The result was a total of 237,306 deletions and 42,767 duplications as a project-level VCF. The length distribution and allele frequency of the project-level VCF are given in Figures 3B,C. Lengths of deletions were presented by using negative values that were shown on the left panel of Figure 3B, while lengths of duplication were shown on the right panel of Figure 3B.

### CNV Concordant Check with Other Projects

We compared our project-level callset with the 1,000 Genomes Project Phase 3 (1KG_P3) (Sudmant et al., 2015), gnomAD (Collins et al., 2020), and Decipher (Firth et al., 2009) that were obtained from dbGaP (https://www.ncbi.nlm.nih.gov/dbvar/content/human_hub/). The 1KG_P3 and gnomAD have other types of variants (insertions, inversions, mobile element deletion, and mobile element insertions) in the lists that were not used in the comparison; only autosomal copy number variations were used for the comparison. All lists were converted into the BED format for performing cross-project concordant CNV checks by using BEDTools.

We examined the overlap between our data and other call sets using either a 1bp or 50% overlap. We performed each pair of comparisons twice treating both callsets as the primary in one of the comparisons. As demonstrated in Table 2, each pair of comparisons is asymmetric with different concordance percentages depending upon which callset was the primary (primary callset is the one in the column). 79.9 and 76.3% of our called CNVs were found in gnomAD and Decipher when using at least 1bp overlapping criterion. However, only 39.8% were recalled in the 1KG_P3 callset. GnomAD likewise has a low concordance rate, with only 41%, of CNVs overlapping with the 1KG_P3 callset. Our callset and gnomAD callset have higher similarity and more novel CNVs compared to the 1KG_P3 and Decipher callsets.

**TABLE 2:** CNV concordant checks with the 1,000 Genomes Project Phase 3 (1KG_P3), gnomAD, and Decipher callsets. Each column resents the percentages of CNVs in the callset overlapping with others listed in rows.

At least 1bp overlap					At least 50% overlap				
	Ours (280,073)	1KG_P3 (48,131)	gnomAD (188,842)	Decipher (54,422)		Ours (280,073)	1KG_P3 (48,131)	gnomAD (188,842)	Decipher (54,422)
Ours	1	0.828	0.762	0.878	Ours	1	0.772	0.726	0.816
1KG_P3	0.398	1	0.410	0.679	1KG_P3	0.293	1	0.337	0.544
gnomAD	0.799	0.861	1	0.832	gnomAD	0.668	0.767	1	0.712
Decipher	0.763	0.662	0.500	1	DECIPHER	0.724	0.600	0.458	1

### CNV List for PLINK Burden Analysis

Since PLINK does not allow overlapping CNVs within a sample, we 1) split the project-level VCF and generated a list of CNVs for a sample in BED format, and 2) consolidated CNVs or removed conflict CNVs by the method described in Section 2.1.4. After splitting the project-level VCF for each sample, we found increased numbers of CNVs per sample (32,402 deletions and 9,131duplications) since GraphTyper2 uses a combination of the three CNV calling algorithms and leverages variant knowledge from other samples. However, most of those CNVs overlap or are adjacent to each other. Next, we consolidated overlapping/adjacent CNVs if they are the same type or removed overlapping CNVs if they are different types. This CNV consolidation step significantly reduces CNVs/sample (2,966 deletions and 1,863 duplications), as shown in Figure 3A.

For rare CNV analysis, we first applied the pilot mask from the 1,000 Genomes Project that further filtered about 8.4% of CNVs and became 2,255 deletions and 1,820 duplications for each sample averagely. CNVs with an allele frequency <1% were retained for analysis. The number of rare CNVs/sample ranged from 0 to 1,546 with an average of 57/sample (46 deletions and 11 duplications; median value is 44 and standard deviation is 76.58843). Among 3,800 samples, three have zero rare CNVs while four have >1,000 rare CNVs. Those four samples are all Non-Hispanic Whites (two cases and two controls), and three of the four samples. According to the final review comment have higher detected numbers of CNVs (According to the final review comment 5,809, 5,945, and 5,992) compared to average (4075.06). The three were sequenced in the earlier stage of the project by Illumina HiSeq 2000/2,500 with PCR Amplified libraries.

### Burdens of All and Rare CNVs

**FIGURE 4:**
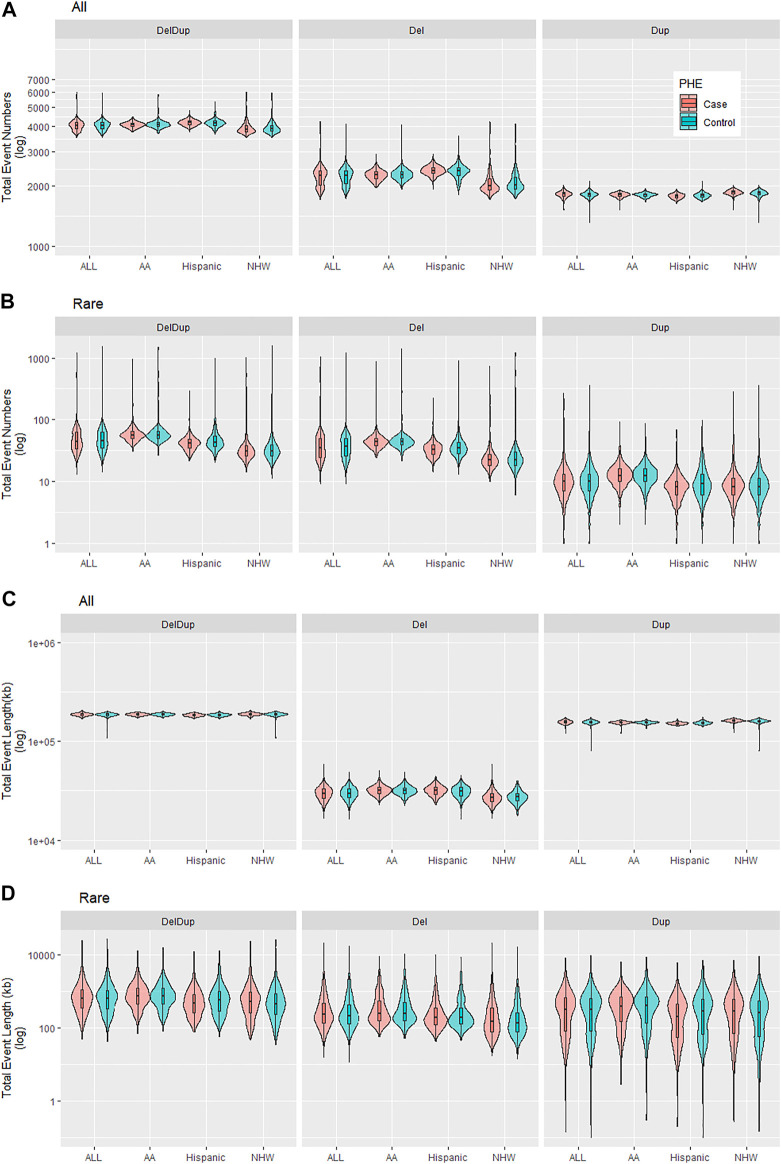
Summary of CNV burden results for all and rare CNVs by CNV events (DelDup, Del, or Dup) and by ethnicities (ALL, AA, Hispanic, NHW). **(A).** Total event numbers per sample. **(B).** Total rare event numbers per sample. **(C).** Total event length in kb per sample. **(D).** Total rare event length in kb per sample.

Table 3 are the PLINK burden tests. The four burden features were considered; 1) total event numbers, 2) Proportion of samples with ≥1 events, 3) total event length in kb, and 4) average event length in kb. Tests were done for all and rare CNVs as well as considering deletions and duplications (DelDup), deletions specific (Del) and duplications specific (Dup). The results suggested two significant all-CNV burden differences between cases and controls: 1) in NHW, on average cases have 16 more duplication events compared to controls do (*p*-value 2e-6); and 2) in Hispanic, the total deletion lengths in cases is larger than in controls on average (*p*-value 6.8e-5). There are no significant differences for rare CNV burden in all aspects examined. Of note, the *p*-values from PLINK burden analysis did not account for covariates and were merely examining if the observed burden measures of cases and controls were significantly different in a marginal fashion. Figure 4 shows the total event numbers per sample and the total event length in kb per sample.

**TABLE 3:** The four burden features were considered; 1) total event numbers, 2) Proportion of samples with ≥1 events, 3) total event length in kb, and 4) average event length in kb.

Mean_Case Mean_Control *p*-value		DelDup		Del		Dup	
		All	Rare	All	Rare	All	Rare
Total event numbers	All	4,073	59.29	2,249	47.67	1823	11.62
		4,079	55.61	2,261	44.41	1818	11.2
		0.736247	0.0709259	0.876096	0.0723559	0.021826	0.132316
	AA	4,072	60.24	2,268	46.81	1803	13.43
		4,106	63.23	2,295	49.7	1811	13.53
		0.990162	0.743957	0.989694	0.753128	0.882104	0.578805
	Hispanic	4,193	42.63	2,408	33.98	1785	8.654
		4,177	59.35	2,384	48.04	1793	11.31
		0.108108	1	0.016028	1	0.974318	1
	NHW	3,991	45.33	2,129	34.1	1861	11.23
		3,972	38.81	2,127	29.01	1845	9.8
		0.158684	0.0287979	0.461645	0.0303239	2e-06*	0.0354999
Proportion of samples with ≥1 events	All	0.9988	0.9988	0.9988	0.9988	0.9988	0.9988
		0.9995	0.9995	0.9995	0.9995	0.9995	0.9985
		0.904246	0.905054	0.905188	0.905048	0.904122	0.581927
	AA	0.9956	0.9956	0.9956	0.9956	0.9956	0.9956
		1	1	1	1	1	1
		1	1	1	1	1	1
	Hispanic	1	1	1	1	1	0.9981
		0.9986	0.9986	0.9986	0.9986	0.9986	0.9986
		0.583197	0.583439	0.582637	0.582109	0.583935	0.826018
	NHW	1	1	1	1	1	1
		1	1	1	1	1	0.9975
		1	1	1	1	1	0.269673
Total event length in kb	All	1.856e+05	1,053	2.983e+04	546.2	1.558e+05	507.2
		1.852e+05	941.4	2.974e+04	457.1	1.555e+05	484.8
		0.017098	0.01129	0.254809	0.00759198	0.0602339	0.148482
	AA	1.859e+05	1,013	3.185e+04	502.7	1.54e+05	510.8
		1.857e+05	1,055	3.175e+04	477.6	1.54e+05	577.4
		0.318897	0.704127	0.330257	0.291605	0.409045	0.942028
	Hispanic	1.83e+05	750.3	3.183e+04	408.3	1.511e+05	342.7
		1.837e+05	911.8	3.097e+04	392.5	1.527e+05	519.4
		0.982962	0.989972	6.79999e-05*	0.356709	1	0.999998
	NHW	1.873e+05	943.1	2.725e+04	455.1	1.601e+05	487.9
		1.863e+05	713.7	2.734e+04	301.8	1.59e+05	412.9
		0.000591999	0.00145	0.670983	0.00347599	0.000116	0.013062
Average event length in kb	All	45.74	19.02	13.34	12.54	85.34	40.48
		45.57	17.96	13.24	11.43	85.47	40.56
		0.0489579	0.0469619	0.0544019	0.0573059	0.995478	0.523385
	AA	45.5	16.3	14.03	10.58	85.04	36.11
		45.29	16.09	13.89	9.459	85.01	38.94
		0.0487079	0.384237	0.108808	0.0501099	0.362197	0.935694
	Hispanic	43.67	16.67	13.26	11.59	84.68	35.79
		44	15.33	13.02	9.041	85.03	39.45
		0.9966	0.0739319	0.00848998	0.00603599	0.999998	0.966586
	NHW	47.31	20.6	12.98	12.82	85.98	41.66
		47.16	18.64	13.02	10.99	86.16	39.69
		0.22001	0.0258339	0.645417	0.0592879	0.98735	0.161868

Tests were done for all and rare CNVs as well as considering deletions and duplications (DelDup), deletions specific (Del) and duplications specific (Dup). Each cell has three values as mean of cases, mean of controls, and *p*-value. Two *p*-values marked in bold indicate statistically significant.

## Discussion

We have composed a scalable bioinformatics pipeline to identify CNVs using WGS data and applied it to 1,737 AD cases and 2,063 cognitively normal controls from the ADSP. We observed 237,306 and 42,767 deletions and duplications, respectively with an average of 2,255 deletions and 1,820 duplications per subject. Although there were more and longer CNVs in AD case samples than controls, burden tests performed using all CNVs or rare CNVs (i.e., <1% in frequency) do not indicate a significant association with AD status.

The false discovery rate of detected CNVs remains uncertain despite the fact that CNVs were generated circumspectly and have been cross checked with other projects including the 1KG, gnomAD and Decipher. The callset of 1KG is smaller than ours and gnomAD’s, and it is therefore expected that 1KG recalls only ∼40% of ours and gnomAD’s callsets, while ours and gnomAD’s callsets capture 82.8 and 86.1% of 1KG’s CNVs respectively. We would also like to note that 1KG processed their data several years earlier than we and gnomAD did. Since the publishing of the 1KG Phase3 callset, CNV-calling tools have moved towards integration of multiple alignment signals (such as read-depth, pair-end, and split-read signals) for calling. This concept was well-accepted before the publishing of the gnomAD callset, and could make 1KG’s callset less similar to ours and gnomAD’s. While extensive experimental validation of each CNV is not currently feasible, validation of significant deletions and duplications may be necessary. Alternatively, our findings could be replicated with other datasets of Alzheimer’s Disease whole genome sequence data.

Joint genotyping provides the ability to leverage information from multiple samples so we could refine low-quality genotypes and detect additional variants for a sample. However, it also brings challenges when breakpoints of CNVs from different samples do not align well. The situation is even worse when using multiple calling algorithms. For this study, we employed GraphTyper2 for joint genotyping, which is a graph-genome based method and has shown an advantage for genotyping larger variants such as CNVs. However, GraphTyper2 does not provide a total solution; overlapping CNVs can still be found after joint genotyping. To address the issue, we split aggregated results to generate a CNV list for each sample and resolved overlapping CNV regions. A graph reference genome presents a variant, a CNV in our application, as a branch in the graph. For the overlapping CNV situation, the graph genome creates several similar branches in a region. The issues could be resolved in a more fundamental way by pruning unnecessary brunches of the graph genome. A slim graph genome will also improve running time and memory usage.

## Data Availability

The data analyzed in this study is subject to the following licenses/restrictions: Data is accessible from NIAGADS DSS *via* qualified access. Formal requests to access these datasets can be submitted to NIAGADS DSS: https://dss.niagads.org/.
