# Supplementary material for: Copy Number Variation Identification on 3,800 Alzheimer’s Disease Whole Genome Sequencing Data from the Alzheimer’s Disease Sequencing Project
Source: Front Genet. 2021 Nov 4;12:752390. doi: 10.3389/fgene.2021.752390 (PMC8599981; doi:10.3389/fgene.2021.752390)
Supplement: Supplementary file 1 [file DataSheet1.docx]

Copy Number Variation Identification on 3,800 Alzheimer’s Disease Whole Genome Sequencing Data from the Alzheimer’s Disease Sequencing Project

**Wan-Ping Lee^1,2,3,*,†^, Albert A. Tucci^4,†^, Mitchell Conery^5,6^, Yuk Yee Leung^1,2,3^, Amanda B. Kuzma^1^, Otto Valladares^1^, Yi-Fan Chou^1^, Wenbin Lu^7^, Li-San Wang^1,2,3^ Gerard D. Schellenberg^1,3^ and Jung-Ying Tzeng^4,7,*^**

^1^Penn Neurodegeneration Genomics Center, Department of Pathology and Laboratory Medicine, Perelman School of Medicine, University of Pennsylvania, Philadelphia, PA, USA

^2^Institute for Biomedical Informatics, Perelman School of Medicine, University of Pennsylvania, Philadelphia, PA, USA

^3^Department of Pathology, Perelman School of Medicine, University of Pennsylvania, Philadelphia, PA, USA

^4^Bioinformatics Research Center, North Carolina State University, Raleigh, NC, USA

^5^Division of Human Genetics, Children’s Hospital of Philadelphia, Philadelphia, PA, USA

^6^Graduate Group in Genomics and Computational Biology, Perelman School of Medicine, University of Pennsylvania, Philadelphia, PA, USA

^7^Department of Statistics, North Carolina State University, Raleigh, NC, USA

**^†^**These authors have contributed equally to this work and share first authorship.

*** Correspondence:**

Wan-Ping Lee and Jung-Ying Tzeng

Wan-Ping.Lee@PennMedicine.upenn.edu and jytzeng@ncsu.edu

**Keywords: copy number variation, Alzheimer’s disease, whole-genome sequencing**

1. Supplementary Data
   1. Script: CNVnator

REF=$WD/GRCh38_full_analysis_set_plus_decoy_hla.fa

BAM=$WD/$SAMPLE.cram

OUT_PATH=$WD/cnvnator/$SAMPLE

CNVnator=$CNVNATOR_PATH/cnvnator

TOVCF=$CNVNATOR_PATH/cnvnator2VCF.pl

BIN=1000

# Extract read mapping

$CNVnator -root $OUT_PATH/$SAMPLE.root \

-tree $BAM -genome GRCh38 -chrom $(seq -f 'chr%g' 1 22) chrX chrY

# Generate read depth histogram

$CNVnator -root $OUT_PATH/$SAMPLE.root -his $BIN

# Calculate statistics

$CNVnator -root $OUT_PATH/$SAMPLE.root -stat $BIN

# Read signal partitioning

$CNVnator -root $OUT_PATH/$SAMPLE.root -partition $BIN

# CNV calling

$CNVnator -root $OUT_PATH/$SAMPLE.root -call $BIN > $OUT_PATH/$SAMPLE.call

# Convert to VCF

$TOVCF $OUT_PATH/$SAMPLE.call > $OUT_PATH/$SAMPLE.vcf

- 1. Script: JAX-CNV

REF=$WD/GRCh38_full_analysis_set_plus_decoy_hla.fa

KMER=$WD/GRCh38_full_analysis_set_plus_decoy_hla.kmer

BAM=$WD/$SAMPLE.cram

OUT_PATH=$WD/jaxcnv/$SAMPLE

JAXCNV=$JAXCNV_PATH/bin/JAX-CNV

$JAXCNV GetCnvSignal \

-f $REF \

-k $KMER \

-o $OUT_PATH/$SAMPLE.bed \

-b $BAM \

--chrom $(seq -s "," -f "chr%g" 1 22),chrX,chrY \

2> $OUT_PATH/$SAMPLE.stderr

- 1. Script: Smoove

https://github.com/hall-lab/speedseq/blob/master/annotations/exclude.cnvnator_100bp.GRCh38.20170403.bed

REF=$WD/GRCh38_full_analysis_set_plus_decoy_hla.fa

BAM=$WD/$SAMPLE.cram

OUT_PATH=$WD/smoove/$SAMPLE

SMOOVE=$SMOOVE_PATH/Smoove

EX_BED=$WD/exclude.cnvnator_100bp.GRCh38.20170403.bed

threads=1

$SMOOVE call --name $SAMPLE \

--outdir $OUT_PATH \

--exclude $EX_BED \

--fasta $REF \

-p {threads} \

--genotype $BAM

- 1. Script: svimmer for a sample

cnvnator_vcf=$WD/cnvnator/$SAMPLE/$SAMPLE.vcf

jaxcnv_vcf=$WD/jaxcnv/$SAMPLE/$SAMPLE.vcf

smoove_vcf=$WD/smoove/$SAMPLE/$SAMPLE.vcf

OUT_PATH=$WD/svimmer/$SAMPLE

SVIMMER=$SVIMMER_PATH/svimmer

for CHR in $(seq 1 22)

do

python3 $SVIMMER $cnvnator_vcf $jaxcnv_vcf $smoove_vcf chr$CHR \

> $OUT_PATH/$SAMPLE.chr$CHR.vcf

done

- 1. Script: svimmer for all samples

OUT_PATH=$WD/svimmer/all_samples

SVIMMER=$SVIMMER_PATH/svimmer

for CHR in $(seq 1 22)

do

VCFS= $(ls $WD/svimmer/*/*.chr$CHR.vcf | paste –sd’ ’)

python3 $SVIMMER $VCFS | bgzip –c > $OUT_PATH/chr$CHR.vcf.gz

done

- 1. Script: GraphTyper2

#Define locations

SV_DIR=$WD/svimmer/all_samples

CRAM_DIR=$WD/cram_files

OUT_PATH=$WD/graphtyper

GRAPHTYPER=$ GRAPHTYPER_PATH/ graphtyper

#Define files

REF=$WD/GRCh38_full_analysis_set_plus_decoy_hla.fa

VCF=$SV_DIR/chr$CHR.vcf.gz

SAM=$CRAM_DIR/cram.list

$GRAPHTYPER genotype_sv \

$REF $VCF --max_files_open 4096 \

--sams=$SAM \

--threads=36 \

--region=$REGION

--output $OUT_PATH

- 1. Script: BED for each sample

VCF_PATH=$WD/graphtyper

DIR=$WD/results

mkdir -p $DIR/$SAMPLE

for i in $(ls $VCF_PATH/*.vcf.gz)

do

j=$(basename $i)

bcftools view -Oz -o $DIR/$SAMPLE/$j -f "PASS" -c 1 -s $SAMPLE $i

done

bcftools concat -Oz -o $DIR/$SAMPLE.vcf.gz $DIR/$SAMPLE/*.vcf.gz

zgrep -v "^#" $DIR/$SAMPLE.vcf.gz \

| grep "AGGREGATED" \

| grep ":PASS:" \

| awk -F'\t' '{print $1,$2,$10,$8}' \

| sed "s/;/ /g" \

| awk '{size=0; type="NA"; for (i=4;i<=NF;i++) {if ($i ~"^SVSIZE=") size=$i; else if ($i ~"^SVTYPE=") type=$i;} print $1,$2,size,type,$3}' \

| sed "s/SVSIZE=//g" | sed "s/SVTYPE=//g" \

| sed "s/:/ /g" \

| awk -v c=$SAMPLE -v OFS='\t' '{id=id+1; print $1,$2,$2+$3,"GraphTyper"id,c,$4,"GraphTyper",$5}' \

> $DIR/$SAMPLE.ori.bed

rm -rf $DIR/$SAMPLE

- 1. Script: CNV segment merging and removing

The BED for telomeres and centromeres is converted from https://github.com/dellytools/delly/tree/master/excludeTemplates.

bedtools=$BEDTOOLS_PATH/bin/bedtools

DIR=$WD/results

BED=$WD/results/$SAMPLE.ori.bed

sed "s/DEL/-/g" $BED | sed "s/DUP/+/g" \

| bedtools merge -i stdin -s -c 6,8 -o distinct,distinct \

| bedtools merge -i stdin -c 4,5 -o distinct,distinct \

| awk -F '\t' -v OFS='\t' -v c=$SAMPLE '{if ($4!="+,-") { type=1;

if ($5=="0/1") type=1; else if ($5=="1/1") type=2;

if ($4=="+") type=2+type; else if ($4=="-") type=2-type;

if ($5 != "./.") print $1,$2,$3,c,type}}' \

> $DIR/$SAMPLE.bed

#Exclude CNVs in telomeres and centromeres.

bedtools intersect -F 0.9 -v -wa -a $DIR/$SAMPLE.bed -b $EXCLU_BED \

| awk -F '\t' -v OFS='\t' '{print $4,$4,$1,$2,$3,$5,0,0}' \

> $DIR/$SAMPLE.cnv

rm $DIR/$SAMPLE.bed

- 1. Script: CNV burden test

PLINK1.07 \

--cfile data \

--cnv-indiv-perm \

--noweb \

--mperm 500000 \

--out data
